# Perceptions and outcomes of an embedded Alzheimer Society First Link Coordinator in rural primary health care memory clinics

**DOI:** 10.1186/s12913-024-11066-0

**Published:** 2024-05-09

**Authors:** Debra G. Morgan, Julie Kosteniuk, Melanie Bayly

**Affiliations:** 1 Canadian Centre for Rural and Agricultural Health, University of Saskatchewan, 104 Clinic Place, Box 23, Saskatoon, SK S7N 5E5 Canada; 2 Research Ethics Office, Human Ethics, University of Saskatchewan, 2nd Floor, Thorvaldson Building, 110 Science Place, Saskatoon, SK S7N 5C9 Canada

**Keywords:** Memory clinic, Rural, Dementia, Primary care, Care navigator, Team-based care, Caregiver support

## Abstract

**Background:**

Primary health care has a central role in dementia detection, diagnosis, and management, especially in low-resource rural areas. Care navigation is a strategy to improve integration and access to care, but little is known about how navigators can collaborate with rural primary care teams to support dementia care. In Saskatchewan, Canada, the RaDAR (Rural Dementia Action Research) team partnered with rural primary health care teams to implement interprofessional memory clinics that included an Alzheimer Society First Link Coordinator (FLC) in a navigator role. Study objectives were to examine FLC and clinic team member perspectives of the impact of FLC involvement, and analysis of Alzheimer Society data comparing outcomes associated with three types of navigator-client contacts.

**Methods:**

This study used a mixed-method design. Individual semi-structured interviews were conducted with FLC (*n* = 3) and clinic team members (*n* = 6) involved in five clinics. Data were analyzed using thematic inductive analysis. A longitudinal retrospective analysis was conducted with previously collected Alzheimer Society First Link database records. Memory clinic clients were compared to self- and direct-referred clients in the geographic area of the clinics on time to first contact, duration, and number of contacts.

**Results:**

Three key themes were identified in both FLC and team interviews: perceived benefits to patients and families of FLC involvement, benefits to memory clinic team members, and impact of rural location. Whereas other team members assessed the patient, only FLC focused on caregivers, providing emotional and psychological support, connection to services, and symptom management. Face-to-face contact helped FLC establish a relationship with caregivers that facilitated future contacts. Team members were relieved knowing caregiver needs were addressed and learned about dementia subtypes and available services they could recommend to non-clinic clients with dementia. Although challenges of rural location included fewer available services and travel challenges in winter, the FLC role was even more important because it may be the only support available.

**Conclusions:**

FLC and team members identified perceived benefits of an embedded FLC for patients, caregivers, and themselves, many of which were linked to the FLC being in person.

**Supplementary Information:**

The online version contains supplementary material available at 10.1186/s12913-024-11066-0.

## Background

 It is estimated that over 55 million people world-wide are currently living with dementia, a number expected to reach 139 million by 2050 [1]. Growth in aging rural populations globally [2], paired with increased risk of dementia with age, means rising numbers of people living with dementia (PLWD) in rural and remote settings. Studies of rural dementia service availability and acceptability report insufficient services and numerous barriers to service use [3–8]. Initiatives in dementia policy and research at the international level [9] and national level in the UK [11, 12], US [13], and Canada [14, 15] emphasize the critical need to address inequities in access to appropriate health and social supports for dementia, including in rural and remote regions. Although policy, research, and practice innovations in rural dementia care are emerging internationally [16], more needs to be done.

Alzheimer Disease International has noted that given rapidly aging populations, current specialist-led models of dementia care are not sustainable or effective, especially in low-resource settings [9]. The report urged a shift toward a model where primary health care (PHC) plays a central role, supporting enhanced early detection and more seamless post-diagnostic care coordination. In Canada, the College of Family Physicians describes family physician roles in key aspects of dementia care including prevention, diagnosis, and post-diagnostic support [17]. Post-diagnostic support is described as the bridge between diagnosis and continuing care that adapts to changing needs [9]. The growing demand for dementia care requires health systems to urgently respond by redefining roles, especially in primary care and in rural areas, and that enhancing PHC for dementia should include expanded roles in case management and service navigation [18].

Research to date supports the need for improved early help with accessing supports for dementia care. Family caregivers often delay seeking help until a crisis, when caregivers are too overwhelmed and unable to continue [19]. A review of rural family caregiver needs in dementia identified needs for improved dementia information and education, better communication with healthcare providers about dementia and its course, and someone to support connections among services [20]. Bayly et al. [3] conducted a review of dementia service availability in rural areas that identified strategies to address service use barriers, including a point of contact to assist in accessing services that matched individual needs. Primary care physicians often lack adequate time and reimbursement systems needed to manage all the complexities of dementia care such as education and care coordination [21, 22].

Dementia care navigation programs have been developed to support the cognitive, functional, behavioral and psychological needs of PLWD and caregivers [23, 24]. Care navigation provides a single point of contact with a key person who offers individualized care management and coordination beginning at the time of diagnosis and over time [25, 26]. Care navigators partner with PLWD and caregivers to identify unmet needs and provide emotional support, coaching, education, skills building, and connection to services [24, 27]. A review of characteristics of navigation programs found that the majority of navigators worked within interdisciplinary care teams [27].

Recent reviews of dementia navigation [28, 29] have identified some positive outcomes related to service use but agree that there is not enough evidence to make substantial conclusions. Giebel et al. [28] conclude that there is mixed evidence on effectiveness of care navigation, despite studies showing sizable benefits for some outcomes, and stress the need for more evaluation of impact on care utilization and in countries outside the USA which does not have universal health coverage. Kokorelias et al. [29] concluded there is strong evidence for care navigation in delaying institutionalization and outcomes such as caregiver mastery, but weak evidence for health service use and functional independence. Both reviews emphasize that although dementia care navigation shows promise, to date evidence of effectiveness is mixed, most studies are US-based and short-term, and the variation in methods, outcome measures, and quality of research indicate the need for more research to more fully understand their impact.

There are also significant gaps in our understanding of the navigator role within primary care memory clinics, and the perceptions of both navigators and team members involved in rural memory clinics. Little is known about the impact of geographic location on navigator roles and functions, how navigators connect with PLWD and caregivers, and how the navigator is situated in the rural care system. The current study describes navigator and clinic team member perspectives of the impact of including a care navigator as a core member of rural primary-care-based memory clinics, and analysis of Alzheimer Society data comparing outcomes associated with different types of navigator-client contacts.

### The Rural Dementia Action Research (RaDAR) program

For over 20 years RaDAR has conducted a community-based participatory research program focused on improving rural health service delivery for PLWD and their families. In 2004 RaDAR launched an interdisciplinary specialist Rural and Remote Memory Clinic at the University of Saskatchewan, Canada [30], initially as a research demonstration project and subsequently funded by the provincial Ministry of Health. Although the clinic mandate is assessment of complex and atypical dementias, we observed that 36% of referrals were for Alzheimer Disease, which Canadian guidelines recommend be diagnosed in primary care [17, 31]. This finding prompted the RaDAR team’s focus on rural PHC for dementia.

An interdisciplinary team-based approach has been described as the most effective way to deliver post-diagnostic support for dementia [31–33]. However, there is a lack of rural-specific models of team-based PHC for dementia aimed at addressing the geographic and resource challenges in settings with sparse populations [34]. Playing a key role in dementia diagnosis and management requires PHC providers to have appropriate education, tools, and supports [9, 13, 14]. To build this needed capacity in PHC, RaDAR established a partnership with one health region in southeast Saskatchewan (Sun Country; population 60,000, area 33,239 km^2^). A regional needs assessment identified three key issues: challenges in early identification and diagnosis, lack of standardized decision support tools to guide assessment, and need for team-based care strategies.

The next step was development of a Rural PHC Model for Dementia [35] based on an extensive scoping review [36]. Seven key elements of best practice were identified and organized into three domains: team-based care (including coordinated care management and education/support for PLWD and caregivers), access to standardized decision support tools and guidelines, and access to dementia specialists for referral and education. Because the scoping review identified little rural-based research, RaDAR then collaborated with one rural PHC team in the Sun Country health region to operationalize model elements in ways that would be feasible, acceptable, sustainable, adaptable to diverse rural contexts, and address challenges identified in the needs assessment. This partnership led to a one-day interdisciplinary primary-care-led memory clinic, held every one to two months, where two patients and their caregivers attend for a half-day each.

The Alzheimer Society of Saskatchewan First Link Coordinators (FLC), who hold a navigator role, were invited to be part of the team to address the components of coordinated care management and education and support for PLWD and families. The Alzheimer Society FLC require a degree in health-related field (social work, kinesiology, nursing, or equivalent), 3–5 years experience with dementia, comprehensive knowledge of the impact of dementia on PLWD and families, and knowledge of community resources. FLC complete comprehensive training on hiring, and annual professional development. More information about the First Link Program is available in Table 1.

**Table 1:** History of the Alzheimer Society First Link Program

• The First Link program was first launched in Canada as a demonstration project by Alzheimer Societies in Ontario and Saskatchewan in 2007 [37]. The program emphasizes strong relationships with primary care, including family physicians and other care team members.
• The key aim is to connect PLWD with education and support as early as possible by encouraging primary care professionals to refer PLWD to First Link at diagnosis, with permission from the PLWD (*direct referral*). The First Link Coordinator (FLC) contacts the PLWD and family directly, and then connects regularly thereafter. FLC offer information about supports and educational opportunities, and provide referral to Society and community-based programs and services throughout progression of dementia. PLWD and family members can also contact First Link themselves (*self-referral*).
• McAiney et al. [37] found that direct referrals to First Link from primary care providers were made sooner after diagnosis compared with self-referrals (mean of 7 months in Ontario and 6 months in Saskatchewan). First Link was a key resource in rural and remote areas where there is limited access to dementia supports and services. The program is now offered across Canadian provinces.

Once the intervention was successfully developed and refined with the first PHC team the clinic model was gradually scaled up to other teams. Eight rural memory clinics have been implemented in mostly small rural communities (range 330 − 11,000 population; median 1305). The RaDAR memory clinics provide diagnosis and ongoing management for community-based individuals with suspected dementia in rural settings. Clinic processes are described in Table 2. Details about clinic development, implementation, sustainability, and scale-up are available in prior publications [35, 38, 39].

**Table 2:** RaDAR memory clinic process

• RaDAR memory clinic teams include a family physician or nurse practitioner lead, occupational therapist, physical therapist, home care nurse or social worker, and a First Link Coordinator (navigator) from the provincial Alzheimer Society. Some clinics include dietician and pharmacy involvement.
• Two new patients and their family members are seen on clinic days for a half day each. For each PLWD-caregiver dyad the assessment begins with a team huddle to review the referral, followed by an initial case conference with the PLWD and family to learn about their concerns and explain the clinic process, individual team member assessments, a team debriefing meeting, and a final team case conference with the PLWD and family to review the findings and recommendations.
• Over the half day the First Link Coordinator meets with the caregiver and participates in the team huddles and case conferences. Note that the term “PLWD” is used in this paper rather than “patient”, although not all are diagnosed with dementia following the assessment.
• Team members’ assessments are guided by the Primary Care Dementia Assessment and Treatment Algorithm (PC-DATA^TM^), a standardized assessment based on Canadian guidelines and adapted for the team approach and electronic medical record use by PHC teams, RaDAR, and PC-DATA developer.
• More information about the clinics is available on the RaDAR website [40].

The objectives of this study were to: (1) examine the First Link Coordinators’ (FLC) perspectives of the benefits and challenges of their role in RaDAR memory clinics, (2) examine the perceptions of other PHC memory clinic team members on inclusion of the FLC in the clinics, and (3) examine differences in client contacts with the Alzheimer Society FLCs between PHC memory clinic clients, direct-referred clients, and self-referred clients.

## Methods

### Study design

This study was conducted with a parallel mixed method design, where qualitative and quantitative data are collected and analyzed concurrently [41] for the purpose of expanding the breadth of the inquiry and enhancing the credibility and integrity of the findings [42]. As a member of the Canadian Consortium on Neurodegeneration in Aging [43] we include sex and gender analyses where feasible.

### Study setting

This research took place in the western Canadian prairie province of Saskatchewan (population 1,132,505, area 577,060 km^2^, density 2.0 persons/km^2^, 34% living in rural areas with less than 10,000 population) [44]. The number of people with dementia in Saskatchewan is expected to more than double in the next 30 years, reaching 42,300 by 2050 [14]. With an aging rural population in the province [45] many of these individuals will be living in rural communities. A local steering group of health region managers, the Alzheimer Society of Saskatchewan, and RaDAR researchers was established in the Sun Country health region in 2013 and continues to meet three times a year. The region is now part of the larger Saskatchewan Health Authority, which provided operational approval.

### Study population

Participants included all FLC coordinators (*n* = 3, all female) who covered the five teams in operation at the time of the study, and members of the memory clinic teams who agreed to participate in the study (*n* = 6, all female).

### Recruitment and consent procedures

FLC and team members were sent individual email invitations and asked to contact the study team to schedule an interview. Consent forms were emailed prior to the interviews, which were audio recorded (including verbal consent), transcribed verbatim, and checked for accuracy. The University Behavioral Research Ethics Board approved the study. The board is governed by the Tri-Council Policy Statement: Ethical Conduct for Research Involving Humans (TCPS 2), a joint policy of Canada’s three federal research agencies.

### Data collection and analysis

#### Qualitative data

Individual semi-structured telephone interviews were conducted in August and September 2020 with the 3 FLC. Between January 2021 and April 2023, 6 members of the memory clinic teams were interviewed by telephone (4) and in-person (2). Interview guides are available in Additional File 1 (FLC) and Additional File 2 (team members). For each set of interviews (FLC, team members) a separate inductive thematic analysis [46] was conducted. Initial coding and preliminary identification of broader themes was carried out independently by two co-authors (DM, JK), who then met to refine the themes for each data set. The third co-author (MB) reviewed the final themes. All authors are doctorally prepared and experienced in rural dementia care research.

#### Quantitative data

A longitudinal retrospective analysis was conducted with previously collected, de-identified records of patients and family members from the Alzheimer Society of Saskatchewan First Link client database between December 2017 and September 2022. Clients seen in the RaDAR memory clinics were compared to self- and direct-referred clients in the same geographic area as the memory clinics on several outcomes: sex, days between referral and first contact, method and duration of first contact, days between contact 1 and 2, total number of contacts, number of completed contacts, number of topics discussed at contact 1, and number of Alzheimer Society and community services recommended at first contact.

Data are presented as N (%) for categorial variables and mean ± SD for continuous variables. Statistical analyses were conducted using ANOVA for continuous variables and Pearson’s Chi-squared test for categorical variables. Analyses were conducted with SPSS version 28.0 [47]. Fisher’s Exact test for categorical variables was used when the expected values in one or more cells was less than 5. The nonparametric Kruskal-Wallis test was used when tests of normality were significant for continuous variables.

## Results

### First link coordinator interviews

The three FLC who covered the five rural memory clinic teams in operation at the time of the study participated in the interviews, which ranged from 24.0 to 45.5 min (*M* = 37.7). All participating FLC were female, which is representative of the Alzheimer Society FLC in Saskatchewan (10/11 female). Each FLC covered 2 to 5 of the memory clinics over the study period. Five main themes were identified: (1) The FLC role, (2) Perceived benefits to patients and families, (3) Benefits to the memory clinic and team members, (4) Benefits to the Alzheimer Society and FLC, and (5) Benefits and challenges experienced by FLC participating in the clinics. Quotations are used to illustrate themes, numbered by the order in which the interviews were conducted (FLC1, FLC2, FLC3).

#### First link coordinator role in the rural memory clinics

The FLCs actively participated in the initial team huddle, initial case conference (with the team, patient, and family), interview with the family that included some time alone with them and some shared time with the homecare nurse or social worker, end of day team huddle, and final case conference. During their time with the family, the FLC learned about their concerns and questions, identified areas where support and other planning were needed, and discussed the frequency of follow-up.

The FLC described their unique role in providing emotional support for family members, and practical information about resources and services available from the ASOS and community programs. This included information on topics such as specific dementia diagnoses, support groups, and education programs, as well as suggestions for handling challenges such as responsive behaviors, memory loss, and driving cessation.

*“It was always the trying to help them with the emotions of it. Like the doctor and the OT and the PT could provide the facts, but it was my role to get more to the emotions and the grief and the loss of it… and honoring that…. ‘I know this is hard to hear… but you know, we can work with you to come up with some ideas to x, y, and z, right? To get through this.’”* FLC3

The aim of the First Link program is to connect with PLWD and family members as soon as possible after diagnosis (or at the time of diagnosis in the memory clinics) to provide ongoing post-diagnostic support through regular, planned contacts.

*“Usually when you’re the care partner of someone with dementia there’s emotional baggage…so people just … chat about how tired they are or whatever. I try to support them that way… we talk about maybe suggestions of how to handle things, like responsive behaviors, or like hey, use a whiteboard next time you go to the garage…. I try to offer some practical suggestions.”* FLC1

#### Perceived benefits to patients and families of FLC involvement in the rural memory clinics

The advantages of the one-day team-based model for patients and families included a faster assessment process, more convenience, a better experience than sequential assessments, and the fact that team members can share information with each other in person. Families are grateful to have the support of someone who understands dementia and the disease process. Importantly, the face-to-face contact at clinic day establishes a relationship that facilitates FLC follow-up contacts and increases the probability that the patient and family will stay connected to the Alzheimer Society and receive needed support in the future. Engaging with PLWD and families is easier in person than on the telephone where subtle nuances can be missed and body language is not accessible.

*“I think it just gives them more comfort. When a doctor or homecare or whoever refers them [direct referral to Alzheimer Society]… it’s just kind of a blind call. Whereas if they met us at clinic… then there’s more comfort there, and more confidence there. They’ve already shared a big piece of themselves with you… there’s that comfort in knowing that we know the story.”* FLC3

The FLC noted that there are advantages to not being members of the provincially funded healthcare system, such as being able to connect people to a wider scope of services and provide encouragement to PLWD and families having difficulties accessing services. Because the FLCs travel out to the clinics in smaller rural centres, they are not members of those communities and are able to see situations from a different viewpoint. Being “outsiders” can address concerns of PLWD and families about confidentiality and add credibility to the FLC role.

*“We’re not only outsiders of the health region, but for the most part outsiders of the community… they’re not going to see you at the rink, they’re not going to see you at the grocery store. And in small towns that kind of matters, right? … I think that the Alzheimer’s Society role was seen as a value add, if you will.”* FLC3

There were advantages to seeing PLWD and their families in their own community, in a familiar clinic environment where they would normally go for medical appointments, by healthcare providers they may already know. This situation was seen as allowing for a more accurate assessment because it was more comfortable and less stressful for the PLWD and family, and local supports were more apparent when teams were delivering the service in the patient’s community.

*“For us to see people in …. that familiar environment, it just gave us a better sense of life… the supports that were in the community were maybe more apparent because we were there.”* FLC3

#### Benefits to the memory clinic and team members of FLC involvement

Like other team members, the FLC have a unique role, allowing other team members to focus on their specific contribution without having to provide the support aspect as well. The complementary roles free up other team members to focus on what they do best.

*“So if dementia wasn’t it [their specialty] it was nice for them to have somebody to refer people to right? So it wasn’t just on them.”* FLC2

The FLC served as a resource to other team members who did not regularly work with PLWD and families and therefore may not be as knowledgeable about dementia subtypes or the kinds of challenges that family members can experience. The FLC shared information they learned from their meeting with the family, which the team may not otherwise be aware of.

*“A lot of the staff would admit they didn’t have a whole lot of dementia information. So that was what I was there for, I was to be the expert in providing that information and support to families specifically in relation to Alzheimer’s Disease and related dementias… So staff even told me after meetings that they had learned from what I said, because they weren’t aware of some of the things people were going through.”* FLC2

Involvement of the FLC in the clinics allowed other team members to see what the FLC role was and what the ASOS could offer to PLWD and families, making it more likely that they would refer non-clinic PLWD to the First Link program.

*“Someone who’s already part of the team would be more likely to fill out the paperwork [to refer to Alzheimer Society]… .because they’ve met me, we have that relationship… it opens that door professionally, and validates the Society’s position and ability to support our clients.”* FLC1

#### Benefits to the Alzheimer Society and First Link Program of FLC involvement in the memory clinics

A major advantage of the memory clinics is the ability of FLC to meet the PLWD and family in person versus making contact on the telephone, which is how the first contact occurs for direct and self-referrals to the Alzheimer Society. The in-person contact enables FLC to better understand the family’s experiences and provide emotional support.

*“I think just that face. Like, putting a face to it… a lot of communication being so nonverbal, I think that being there talking with them, doing a touch—you know, a touch on the knee or a touch on the shoulder, handing them a Kleenex… just being able to support that person in person means a lot.”* FLC1

The involvement of FLC in the clinics opens doors professionally by fostering relationships with other team members, so that FLC are more comfortable contacting team members about clinic patients if new needs emerge during FLC follow-up appointments. Another benefit of FLC involvement is learning about the PLWD and family from other team members, most of whom are local and know the PLWD and family.

*“In the memory clinic I find it’s a little bit easier, because I might have heard the doctor say something, and I can pull from other people’s assessments. You know, when we’re in a small town like [XXX] these people… know the clients, from like forever!… with most of my direct referrals or self-referrals, they’re coming in blank to me. Like I don’t know who they are, so I can’t pull any of that extra stuff out, like it’s harder”* FLC1

The sharing of knowledge among team members exposed the FLC to perspectives and roles of different disciplines in relation to dementia care and resulted in mutual learning. The FLC found it rewarding working as part of the team and feeling they are making a difference for PLWD and families. All described the shared learning and teamwork as a very positive experience and valuable for everyone.

*“I really wish all our FLC could have this experience… there’s so much value in these memory clinics, in being part of the memory clinic, and then the shared knowledge that we get, working with, you know, the physiotherapist, and OT, and the doctor.”* FLC3

The FLC felt accepted and respected as part of the team from the outset. Being involved in the clinic raised awareness of the ASOS and FLC contributions to dementia care, and confirmed and elevated the importance of their role. They appreciated how committed all team members were to the common goal of better care for PLWD and families in rural settings.

*“I think it really validates the Alzheimer Society’s place in the memory clinic. ‘Cause I’m the only unregistered person… the only one who is outside the health care system as well… I think it really ups our… like, position. So I feel really honored that I get to be there, that my voice is heard and my suggestions are validated.”* FLC1

Finally, being in the rural communities for the clinics was an opportunity for FLC to do outreach and to increase exposure of the Alzheimer Society, by visiting hospitals, health centres, and physician offices to drop off Society brochures and resources.

#### Benefits and challenges associated with FLC involvement in the rural memory clinics

FLC viewed the rural location of the memory clinics as a benefit to patients and families, who no longer had to travel to urban centres for assessment. A challenge for the FLC was the limited number of post-diagnostic resources and supports in rural communities for them to make referrals to. Also, not everyone was ready to accept support.

*“If anybody understands rural… there’s not a lot of support… there’s not a lot of anything out in these rural communities. [Clinic community] is in the middle of nowhere, it’s two hours away from everything basically… people would have to drive to [city] to the [urban] memory clinic to get assessed, and wait however long they had to wait. So there was huge benefits to bringing it to the people in these rural communities.”* FLC2

The FLC often had to travel long distances to the clinics and were sometimes unable to attend in person due to winter driving conditions. During the COVID-19 pandemic the FLC could only attend virtually, which limited their ability to engage with patients and families. Although the Alzheimer Society adapted many of their education and support programs to virtual delivery, not everyone had on-line access.

*“Like with Covid, we’ve changed how we’re rolling… I’ll do it remotely, my portion, I’ll be a face on the screen. And I’m happy to do that [but] I think it will take away a little bit from the experience, just those subtle nuances when you’re together in a room and you can feel someone’s body language.*” FLC1

### Primary health care team member interviews

Six team members participated in interviews exploring their perceptions of the FLC role in the rural memory clinics. All participants were female, which is representative of the teams as across the five clinics only two active team members were male at the time of the interviews. Interviews ranged from 5.7 to 16.4 min (*M* = 10.3). Three key themes were identified: (1) Perceived benefits to patients and families of FLC involvement, (2) Benefits to the memory clinic and team members of FLC involvement, (3) Impact of rural location on FLC coordinator involvement. Quotes from team members are numbered by the order in which the interviews were conducted (e.g., TM1, TM2).

#### Perceived benefits to patients and families of FLC involvement in the rural memory clinics

All participants noted the key role of the FLC in providing education and resources to PLWD and families about the different types of dementia, the Alzheimer Society programs and services available to them, and other community services that may be useful. The emotional support provided by the FLC was also identified as a unique role on the team.

*“You especially see that [impact] with the family who’s struggling as a caregiver; struggling with their own emotions around it. The FLC is a huge resource for them in realizing what they’re going through and just being present for them; giving them that sense of belonging that there’s no shame in what they are going through or what they’re going through is normal…. I can’t say enough good things.”* TM6

The FLC role is focused on the caregiver and their concerns, whereas other team members are involved in assessment of the PLWD. Team members emphasized that without FLC involvement, caregiver needs would not be taken into account in the same way.

*“They have the time to talk and listen to the family and hopefully make sure all the family’s concerns are being heard. And they can also provide resources to the family for how best to help the person with dementia. And they have lots of resources for caregivers, care of themselves.”* TM3

*“[The biggest benefit is] the relationship and the engagement with the Alzheimer’s Society and their ability to have a connection—the patient’s ability to have connection to support.”* TM6.

Being in person at the clinic makes the FLC role more effective because they are able to establish a relationship with the caregiver, compared to self or direct referrals where contact is by telephone.

*“It’s nice to have a face attached to an organization. It’s one thing for us to make a referral to First Link, to the Alzheimer’s Society, but it’s a whole other thing if there’s an actual face that’s sitting around the room…. it becomes more personalized and… a little bit less scary for them.”* TM4

*“We can do assessments, we can do treatment plans, but the supportive resources, the emotional resources, the social support, the education sessions…. the FLC is the direct connection to that. We could give out that information… but you don’t get the same engagement… the same buy-in when people are able to see the face and know the person on the other end that they’re talking to.”* TM6

At the clinics the FLC meets with the caregiver during their initial assessment, whereas with other types of referrals there is a delay in connecting with the FLC.

*“I find when they’re here [in person] patients get better access to them versus when we refer them, there’s just more lag, they’re not as open with them [when direct referred]. So I find they really do create that relationship to help them feel more supported.”* TM5

The FLC provide regular follow-up after the initial clinic day assessment. The ongoing connection is important as needs can change over time.

*“One of the gaps they fill is they provide a lot of follow-up for the family, after. Even after the clinic they’ll phone and touch base with the family, and then they continually do that.”* TM3

*“It can be helpful if there’s been a diagnosis made…. it allows the individual, as well as the family, to know that there are ongoing supports and resources outside of that clinic. I think it can often give the family a sense of reassurance.”* TM4

#### Benefits to the memory clinic and team members of FLC involvement

The involvement of the FLC benefitted other team members by allowing them to focus on their unique roles, knowing that the FLC are there to support the caregivers, learning about their concerns and how they are managing. The FLC then bring this knowledge to the closing team case conference where recommendations are discussed, giving team members a fuller picture of family functioning and needs.

*“They kind of get [the family’s] perspective of how things are going, which is really helpful, because… then when we have our team meeting, they’re able to bring forward any concerns that maybe other team members didn’t pick up on. And then they are a huge resource for the team in terms of resources and education… They often have lots of good ideas of what’s available in the community for different programs.”* TM3

Another benefit of the FLC participation is greater visibility of the FLC role and increased awareness of the Alzheimer Society’s services and their value. Team members reported that because of their exposure to FLC in the clinic they now refer non-clinic patients to the First Link program.

*“[I am] just definitely more aware of what’s available. And I know why it’s beneficial as well… I can really see the value of it, I guess, now that I see their interaction with families and what they bring.”* TM3

The FLC were a resource to the team, bringing information about Alzheimer Society services such as education programs and support groups that other team members may not be aware of. Driving issues were common and team members appreciated FLCs’ suggestions and resources in managing this difficult topic. Caregivers sometimes felt overwhelmed, and the FLC was helpful in sorting out what to focus on.

*“Yes, absolutely [a benefit to the team] because we don’t know the scope and we don’t offer those same services. In order to make it really team-based and holistic, the FLC are coming in with more of the emotional support, the education tools, and the gaps, the things that the [health region] doesn’t provide.”* TM6

Knowledge about dementia subtypes and their management was another important contribution of the FLC that benefited other team members with less experience in working with PLWD and caregivers.

*“Just bringing that… specialty knowledge about our dementias and different ways they’ll present, and also looking at ways we can support patients and their families [that] we don’t think of, like outside community resources for them.”* TM4

#### Impact of rural location on FLC involvement

It was noted that the FLC role is even more important in rural settings compared to urban locations that have more supports and services. Small centres do not have the capacity to offer local education and support groups, which the Alzheimer Society delivers virtually.

*“Just the fact that they have a lot of virtual resources, they have a lot of online learning sessions…. So we don’t have to have the person right in our community and every small community… education and support groups and all that kind of stuff. I think they’re well-suited to providing support in a rural location.”* TM3

*“I feel there’s probably more benefit with the rural …. when they’re more isolated and there’s less resources—the Alzheimer’s Society definitely fills that gap with their FLC in a much more valuable way than, say, someone in [larger centre] who would have access to counseling … mental health. Those rural areas don’t have that. This might be the only support those families and caregivers have.”* TM6

When asked about the FLC role in rural clinics, team members commented on the value of having the FLC in person, describing their experiences during the COVID-19 pandemic when FLC were unable to travel. Although they participated virtually, team members observed that it was difficult to create the same connection with the FLC.

*“I think it’s a really good thing to have [in rural]… it’ll allow for more continuity with the family to follow through with those supports, reaching out, just having that personal connection.”* TM4

One team member stated that some family members are deterred from engaging with the FLC when they learn the FLC is associated with the Alzheimer Society. She observed that this occurred more often in situations where a specific diagnosis had not yet been made, a non-Alzheimer diagnosis was given, or when the caregiver may not be ready to accept a diagnosis of Alzheimer Disease. This participant also commented that community size can influence the FLC role, with more openness to engaging with the FLC in larger centres. She speculated that perhaps rural people are more wary of people they do not know. Her experience was that in smaller centres people often know the homecare nurses and are more likely to accept help from them.

*“[Clinic location] is city enough that there’s not as much personal connections with the patients. So they’re kind of more willing to go with the outside resource. But [another clinic location], because it’s small, and if they don’t know you they don’t want to talk to you.”* TM2

### Alzheimer Society First Link Client Database

The last component of this study was analysis of the Alzheimer Society of Saskatchewan’s First Link Client database. A total of 139 clients had contact with Alzheimer Society FLCs between Dec 2017 and September 2022 (47 rural memory clinic, 34 self-referred, 58 direct-referred) (Table 3). Client relationship to the PLWD was self (16%), spouse/partner (41)%), adult child (3%), and other family or friend (8%). Females represented 44% of the sample (61/139) although sex data were missing for 44 clients. There were no significant differences in proportions of females and males across the three groups (*p* = 0.313, 44 missing). Mean client age at referral was 69 years (99 missing); mean age of PLWD at referral was 80 years (70 missing).

**Table 3:** Descriptive characteristics and differences in contact numbers, duration, method, number of topics discussed, and number of services recommended for rural memory clinic (RMC), self, and direct-referred First Link clients

Variables	Total *N* = 139	Rural Memory Clinic (RMC) *N* = 47	Self-referred *N* = 34	Direct referred *N* = 58	*p* value
Sex^a^ (*n* = 95)					
Female	61 (64.2)	21 (56.8)	21 (75.0)	19 (63.3)	0.313
Male	34 (35.8)	16 (43.2)	7 (25.0)	11 (36.7)	
Relationship to person with dementia^b^ (*n* = 135)					
Person with dementia	22 (16.3)	14 (30.4)	3 (9.1)	5 (8.9)	0.027
Spouse/partner	55 (40.7)	15 (32.6)	12 (36.4)	28 (50.0)	
Child	47 (34.8)	13 (28.3)	13 (39.4)	21 (37.5)	
Other	11 (8.1)	4 (8.7)	5 (15.2)	2 (3.6)	
Duration of Contact 1^c^ (*n* = 131)					
15–30 min	50 (38.2)	3 (6.4)	15 (46.9)	32 (61.5)	< 0.001
45–90 min	45 (34.4)	8 (17.0)	17 (53.1)	20 (38.5)	
3–4 h	36 (27.5)	36 (76.6)	0	0	
Method of Contact 1^d^ (*n* = 132)					
In person	47 (35.6)	40 (85.1)	4 (12.5)	3 (5.7)	< 0.001
Phone or other	85 (64.4)	7 (14.9)	28 (87.5)	50 (94.3)	
Days between referral to ASOS and Contact 1^e^ (*n* = 130)	5.8 *±* 13.0	1.3 *±* 3.3	6.1 *±* 17.9	9.3 *±* 13.6	< 0.001
Days between Contact 1 and Contact 2^f^ (*n* = 125)	45.0 *±* 49.8	47.2 *±* 36.5	54.7 *±* 56.0	37.5 *±* 55.8	0.012
Total number of contacts^g^ (*n* = 139)	8.0 *±* 5.6	9.2 *±* 5.1	6.9 *±* 6.1	7.7 *±* 5.6	0.046
Number of completed contacts^h^ (*n* = 139)	4.4 *±* 3.2	5.2 *±* 2.7	3.8 *±* 3.7	4.2 *±* 3.3	0.029
Number of topics discussed at first completed contact^i^ (*n* = 131)	9.5 *±* 6.2	11.5 *±* 6.8	8.3 *±* 6.5	8.4 *±* 5.1	0.046
Number of recommended ASOS services at first completed contact (*n* = 131)	2.4 *±* 2.0	2.5 *±* 1.8	2.2 *±* 2.0	2.4 *±* 2.2	0.608
Number of recommended community services at first contact (*n* = 131)	0.5 *±* 0.8	0.6 *±* 1.0	0.3 *±* 0.6	0.4 *±* 0.7	0.150

For categorical variables, n (%) is shown; comparisons across all groups and between each pair of groups were conducted with Pearson’s Chi-squared test or Fisher’s Exact test. For continuous variables, mean *±* SD is shown; comparisons across all groups and pairwise comparisons were conducted with the Kruskal-Wallis test

^a^ RMC v Self (*p* = 0.128); RMC v Direct (*p* = 0.585); Self v Direct (*p* = 0.337)

^b^ RMC v Self (*p* = 0.134); RMC v Direct (*p* = 0.020); Self v Direct (*p* = 0.220)

^c^RMC v Self (*p* < 0.001); RMC v Direct (*p* < 0.001); Self v Direct (*p* = 0.189)

^d^RMC v Self (*p* < 0.001); RMC v Direct (*p* < 0.001); Self v Direct (*p* = 0.417)

^e^ RMC v Self (*p* = 0.459); RMC v Direct (*p* < 0.001); Self v Direct (*p* < 0.001)

^f^ RMC v Self (*p* = 0.576); RMC v Direct (*p* = 0.004); Self v Direct (*p* = 0.052)

^g^RMC v Self (*p* = 0.014); RMC v Direct (*p* = 0.118); Self v Direct (*p* = 0.258)

^h^RMC v Self (*p* = 0.012); RMC v Direct (*p* = 0.049); Self v Direct (*p* = 0.402)

^i^RMC v Self (*p* = 0.035); RMC v Direct (*p* = 0.033); Self v Direct (*p* = 0.807)

Pairwise comparisons between groups are shown at the bottom of Table 3. Client relationship to the PLWD differed between memory clinic and direct referrals (*p* = 0.020) with the PLWD being identified as the client more often than with direct referrals. The duration of contact 1 was significantly longer for memory clinic clients (*p* < 0.001) with 76.6% having a 3–4 h contact with the FLC. The FLC have a scheduled 1-hour meeting with caregivers, participate in initial and final case conferences, and are present throughout the half-day appointment. For self- and direct-referred clients, the duration was more evenly split between 15 and 30 min contacts and 45–90 min contacts. In terms of method of contact, most memory clinic contacts were in-person (85.1%), whereas the majority of self-referred (87.5%) and direct-referred clients (94.3%) were by telephone/other (*p* < 0.001).

There was a significant difference in number of days between referral to the First Link program and contact 1 (*p* < 0.001) with memory clinic clients being contacted sooner than direct-referred clients (*p* < 0.001). The number of days between contact 1 and contact 2 was significantly different, with a longer time between contacts for memory clinic compared to direct-referred clients (*p* = 0.004). The number of completed contacts differed significantly across groups (*p* = 0.029); memory clinic clients had more completed contacts than both self (*p* = 0.012) and direct-referred clients (*p* = 0.049). Across the groups there were significant differences in the number of topics discussed at contact 1 (*p* = 0.046); more topics were discussed with memory clinic clients compared to self (*p* = 0.035) and direct referrals (*p* = 0.033). There were no differences between groups on number of Alzheimer Society or community services recommended at contact 1.

## Discussion

This study examined the role of care navigators (Alzheimer Society First Link Coordinators) in rural-based primary care memory clinics, from the perspective of team members and FLC. The study used a mixed qualitative and quantitative design to highlight the contributions of care navigators embedded within interprofessional memory clinic teams, with a focus on rural settings. There was overlap in key themes across the team member and FLC interviews on the perceived benefits of the FLC role for PLWD and family, and for the team members. The central finding was the essential role of FLC in the memory clinics; while other team members’ assessments were concentrated on the PLWD, the FLC was the only team member primarily focused on the caregivers and family.

The FLC role as characterized by study participants is more than navigation to services. Both the FLC and memory clinic team members spoke about the emotional and psychological support the FLC provide, enabling caregivers to talk about their experiences and concerns in a non-judgemental atmosphere. FLC also provide information on specific dementia subtypes, what to expect in the future, what programs and services are available and how to connect with them, and suggestions for managing memory-related and behavioral symptoms of the PLWD. These findings are consistent with earlier studies describing the importance of initial and ongoing psychological support, education, information, and connection to dementia support services [48, 49], especially in rural settings [3, 5, 25, 50–52] and represent key activities that the dementia care navigator role can be targeted to as a member of an interprofessional team.

Another key finding was their views on the importance of the FLC’s face-to-face contact with PLWD, caregivers, and team members. Team members and FLC emphasized how being in person in the clinic helped to develop a relationship between the caregiver and FLC, which made the experience more personalized, reassuring, and supportive for caregivers, resulting in more engagement. The relationship established at the initial assessment also facilitated future FLC contacts, increasing the probability of a long-term connection with the FLC. A related benefit of in-person presence was the ability of FLC to learn more about the PLWD and caregiver from other team members, which helped in tailoring the care plan developed at the final team case conference. Care coordination and building the client-navigator relationship have been described as the defining characteristics of patient navigation [27]. The benefits of having the FLC embedded in the team highlight the advantages of this approach compared to stand-alone navigation models.

Findings from the FLC and team interviews regarding the benefits of having a FLC as a core team member and attending in-person, were supported by analysis of the Alzheimer Society First Link database. Compared to self- and direct-referrals, in which FLC contacts are conducted by telephone, those seen in the clinics were contacted by a FLC sooner after referral, had contacts that were longer in duration, had more completed contacts, and had more topics discussed with FLC. The longer time between initial and second contact for memory clinic clients may be due to the longer time spent with clients on clinic day and face-to-face interactions that facilitate more in-depth conversations compared to telephone, making the need for a second contact less urgent.

A review of facilitators and barriers to implementation of patient navigator programs found that using referral from physicians to obtain clients could cause recruitment difficulties [27]. Connection with the First Link program can be made directly by PLWD and caregivers (self-referral), but health professionals are encouraged to refer at diagnosis, with permission of the PLWD or caregiver (direct referral). A challenge with the latter approach has been low rates of referral, leading to efforts to promote the program with primary care physicians [37]. Findings from the current study suggest that presence of the FLC in the memory clinics supports the earliest possible connection with the First Link program because of the immediate contact in the clinic, bypassing the need for referral. Early education and connection to supports can delay institutionalization and reduce caregiver distress [3, 37, 52]. Other barriers to delivering navigation programs [27] were difficulties connecting with patients’ primary care providers and partnering organizations, both of which were ameliorated by inclusion of the FLC in the rural clinic teams. Collaboration with key stakeholders by embedding local Alzheimer Society representatives into memory clinics was identified in the review as an implementation facilitator.

Earlier studies have identified essential aspects of support worker roles such as FLC that have shown a positive impact on caregiver burden and quality of life. These include face-to-face contact, initial and ongoing follow-up throughout the course of dementia, individualized education and support based on needs, participation in multi-disciplinary teams, and inter-professional collaborations with a shared approach to care [52]. The presence of a consistent key contact person has been suggested as a strategy to overcome barriers to service use [10, 48], with early contact to provide time to establish a “bond of trust” and understanding of needs [19]. Rural-based studies have identified that a single point of access for information and referral, and a presence in local communities, were important [3, 52]. Including the navigator in the rural memory clinic team is one strategy for ensuring this point of access for PLWD and families at the initial assessment, and facilitating early and ongoing follow-up with the FLC.

The FLC involvement in the clinic benefited team members by allowing them to focus on their roles knowing that caregivers were being supported, and by learning about available community and Alzheimer Society services. Rural family physicians recognize caregivers’ need for education and emotional support and see this as part of their role [21, 53] but like many primary care physicians they lack the resources and time needed to provide ongoing care management and support [22, 24]. Heintz et al. [22] assert that within collaborative care teams, dementia care managers, which share many facets of the FLC role, can ensure that PLWD and caregivers have access to the information and support needed to obtain services, while also improving primary care providers’ work lives and sense of competence. Including an Alzheimer Society representative in memory clinics has been found to reduce burden and stress of team members, who were relieved knowing that education, information, and navigation support were being provided [54].

Both FLC and team members noted challenges associated with the rural location of the clinics, including fewer services for FLC to refer to and travel required for FLC to participate. The limited service options in rural communities was seen as making the FLC role in the clinics even more important than in larger centres, as they provide an ongoing contact and support in the absence of other resources. Other studies have reported on the reduced availability of dementia services in rural settings [7, 20, 25, 27] and the need for better care coordination to help caregivers feel comfortable using services [5, 50]. For FLC, benefits of participation in the rural clinics included the credibility associated with being a community “outsider”, improved understanding of local supports, learning from team members who know clients well, and raising team members’ awareness of the FLC role and Alzheimer Society services.

The practice-oriented service model [5] outlines three types of information that caregivers require to use a support service: believing there is a need, knowing a service is available; and knowing how to obtain it. The current study identified that FLC provide all of these types of information. Recommendations from a review of rural dementia education and service availability [3] included having a point of entry to service use, inter-organizational collaboration, education, and development of person-centred services tailored to individual preferences and needs. Results of the current study indicate that these strategies to counter barriers to service accessibility and use are addressed by having FLC as integral members of rural memory clinics. Our findings are consistent with the recommendation that improving access to services requires dementia care navigators to be well integrated into health and social care systems [28].

### Strengths and limitations

This study has potential limitations. It was conducted with a small number of memory clinic teams within one geographic area of the province where the clinics are currently located. Additionally, it is possible that team members with more positive views of the FLC role may have been more likely to participate in the study. The inclusion of both FLC and team member perspectives, as well as analysis of Alzheimer Society data comparing three types of referrals, is a strength. The consensus between FLC and team members on the perceived benefits of FLC participation reinforces the credibility of these findings. Sex and gender analyses were not possible because all participating FLC and team members were female. This should be a focus for future research, as no existing studies were found on this topic. Across all five clinics included in the study, team members were overwhelmingly female. It is unclear how study results might be different if healthcare providers on the clinic teams were male. The Alzheimer Society database had considerable missing data for age of the client and PLWD, and client sex. However, since April 2023 the Society is regularly collecting data on age, sex, gender, ethnicity, race, and languages spoken and read for those accessing their services, which will facilitate future research on understanding the needs of diverse communities. The absence of involvement of PLWD and caregivers is a limitation. At the time of this study we had several studies ongoing (PLWD and caregiver experiences with the RaDAR memory clinics, support and service needs before and after initial assessment, quality of life), and we were concerned about burdening them with additional data collection. Future research should explore perceptions of PLWD and caregivers attending the clinics specifically regarding FLC involvement.

## Conclusions

Alzheimer Disease International has called for a greater role for primary care in dementia that includes support and care coordination beginning at diagnosis and sustained over time [9] and for post-diagnostic support that is more effective, equitable, and accessible wherever people live [10]. The importance of primary care for dementia is especially critical in rural areas with few specialists and other resources. Embedding the FLC in the clinics helps to addresses the key needs identified by rural caregivers of PLWD for navigation support, reducing the challenges of travel to access services, and addressing the lack of dementia-specific services and expertise in rural settings. The current study shows that the inclusion of a navigator role in rural primary health care memory clinics can address these recommendations, is feasible, and has the potential to benefit PLWD, caregivers, and other team members.

### Supplementary Information

Supplementary Material 1. First Link Coordinator interview guide. This semi-structured interview guide was used for interviews with the three Alzheimer Society First Link Coordinators working with the five RaDAR memory clinics in operation at the time of the study.

Supplementary Material 2. Memory clinic team member interview guide. This semi-structured interview guide was used for interviews with healthcare professionals involved in the five RaDAR memory clinics in operation at the time of the study.

## Data Availability

The datasets generated for this study (interviews with First Link Coordinators and RaDAR memory clinic team members) are not publicly available for reasons of participant confidentiality due to the location of the primary healthcare teams in small rural communities and small sample size, but are available from the corresponding authors on reasonable request. The Alzheimer Society First Link data were obtained under agreement with the Alzheimer Society of Saskatchewan for this study and not publicly available. These data are available from the authors upon reasonable request and with permission from the Alzheimer Society of Saskatchewan.

## Abbreviations

PLWD: People living with dementia
PHC: Primary health care
RaDAR: Rural Dementia Action Research
FLC: First Link Coordinator
