# Supplementary material for: Perceptions and outcomes of an embedded Alzheimer Society First Link Coordinator in rural primary health care memory clinics
Source: BMC Health Serv Res. 2024 May 9;24:607. doi: 10.1186/s12913-024-11066-0 (PMC11080231; doi:10.1186/s12913-024-11066-0)
Supplement: Supplementary file 1 — Supplementary Material 1. First Link Coordinator interview guide. This semi-structured interview guide was used for interviews with the three Alzheimer Society First Link Coordinators working with the five RaDAR memory clinics in operation at the time of the study. [file 12913_2024_11066_MOESM1_ESM.pdf]

Additional File 1

First Link Coordinator Interview Schedule

1. How long were you/have you been part of the rural memory clinics as a First Link Coordinator?

2. Please walk me through what you do when you attend the rural memory clinics.
    a. Is there anything you do to prepare before you see the clients?
    b. Is there anything you do after seeing clients and families to wrap up (e.g., chart, discuss with other team members?)

3. What has your contact with memory clinic clients/families been like after their clinic visit?
    a. Do you usually follow up with them?
    b. How have the follow ups been going?
    c. Have they been contacting you for more information/resources/support?

4. Please describe what it has been like for you being part of the memory clinic teams.
    a. How do you find being part of the team conference at the end of the patient's visit?
    b. How do you feel you contribute to the memory clinics? What is your unique contribution?
    c. How do you think the memory clinics are different as a result of your participation?
    d. Has your experience been different between the different teams? If so, please describe.

5. Have you found anything challenging about your role in the memory clinics? Please describe these challenges.
    a. What do you think would have to change to get rid of these challenges? Are these changes possible?

6. Are your client interactions the same or different through the memory clinic compared to typical interactions with clients (those who are direct-referred or self-referred)?
    a. In terms of establishing rapport, client engagement, consultation content, etc.
    b. Are these differences/similarities the same for later consultations and the initial contact?

7. Do you feel there are benefits to patients and families from having the First Link Coordinator involved in the memory clinics? Please describe these benefits.

8. Do you feel there are benefits to rural memory clinics/PHC teams from having the First Link Coordinator involved? Please describe these benefits.

9. Do you feel there are benefits to the Alzheimer Society/First Link Program from being involved in the memory clinics? Please describe these benefits.

10. Do you feel like you benefit professionally from your involvement in the rural memory clinics?

11. How do you feel the rural location of these clinics changes the benefits of being involved? This could be benefits to you, families, the teams, or the ASOS.

12. How do you feel the rural location of these clinics changes the challenges of being involved in the memory clinics?

13. Is there anything else you would like to say about your experiences with the rural memory clinics?
