# Supplementary material for: Perceptions and outcomes of an embedded Alzheimer Society First Link Coordinator in rural primary health care memory clinics
Source: BMC Health Serv Res. 2024 May 9;24:607. doi: 10.1186/s12913-024-11066-0 (PMC11080231; doi:10.1186/s12913-024-11066-0)
Supplement: Supplementary file 2 — Supplementary Material 2. Memory clinic team member interview guide. This semi-structured interview guide was used for interviews with healthcare professionals involved in the five RaDAR memory clinics in operation at the time of the study. [file 12913_2024_11066_MOESM2_ESM.pdf]

Additional File 2

Interview schedule for memory clinic team members

1. How would you describe the role of the First Link Coordinator in the rural memory clinics?
    a. What are the activities they do during clinics?
    b. How much direct interaction do you have with the Coordinator during the memory clinics?
    c. How do you feel about their participation in the team conferences?
    d. Are there unique gaps this role fills?

2. Do you have any follow up discussions about memory clinic patients with the First Link Coordinator, separate from the clinic day?
    a. For example, do you discuss patient/family uptake of recommendations, or further resources the patient/family may need?

3. Do you feel there are benefits to patients and families from having a First Link Coordinator involved in the memory clinics? Please describe these benefits.
    a. Does the rural location of the clinics change these benefits at all?

4. Do you feel there are benefits to yourself and other clinic team members from having a First Link Coordinator involved? Please describe these benefits.
    a. Do you think the benefits to yourself are the same or different as the benefits to other team members?
    b. Do you feel like your relationship with the Alzheimer Society has changed as a result of a First Link Coordinator being part of the clinics?
        i. Has your knowledge of services and supports in the community changed?
    c. (if applicable) Have you referred non-memory clinic patients to the Alzheimer Society? Have your referral patterns changed because of the First Link Coordinator's involvement in the memory clinics?

5. Have you found anything challenging about having a First Link Coordinator involved in the memory clinics? Please describe these challenges.
    a. What do you think would have to change to get rid of these challenges? Are these changes possible?
    b. Does the rural location of the clinics change these challenges at all?

6. Is there anything else you would like to say about the First Link Coordinator's role in the rural memory clinics?
